# Supplementary material for: Remote solid cancers rewire hepatic nitrogen metabolism via host nicotinamide-N-methyltransferase
Source: Nat Commun. 2022 Jun 15;13:3346. doi: 10.1038/s41467-022-30926-z (PMC9200709; doi:10.1038/s41467-022-30926-z)
Supplement: Supplementary file 1 — Supplementary Information [file 41467_2022_30926_MOESM1_ESM.pdf]

Supplementary Information

Remote solid cancers rewire hepatic nitrogen metabolism via host nicotinamide-N-methyltransferase

Rin Mizuno[1,2], Hiroaki Hojo[1,3,4], Masatomo Takahashi[5], Soshiro Kashio[6], Sora Enya[3,4], Motonao Nakao[5], Riyo Konishi[1], Mayuko Yoda[1], Ayano Harata[1], Junzo Hamanishi[2], Hiroshi Kawamoto[7], Masaki Mandai[2], Yutaka Suzuki[8], Masayuki Miura[6], Takeshi Bamba[5], Yoshihiro Izumi[5], Shinpei Kawaoka[1,3,4,9*]

[1]Inter-Organ Communication Research Team, Institute for Life and Medical Sciences, Kyoto University, Kyoto 606-8507, Japan

[2]Department of Gynecology and Obstetrics, Kyoto University Graduate School of Medicine, Kyoto 606-8507, Japan

[3]The Thomas N. Sato BioMEC-X Laboratories, Advanced Telecommunications Research Institute International (ATR), Kyoto 619-0237, Japan (previous affiliation)

[4]ERATO Sato Live Bio-forecasting Project, Japan Science and Technology Agency (JST), Kyoto 619-0237, Japan (previous affiliation)

[5]Division of Metabolomics, Research Center for Transomics Medicine, Medical Institute of Bioregulation, Kyushu University, Fukuoka 812-8582, Japan

[6]Department of Genetics, Graduate School of Pharmaceutical Sciences, The University of Tokyo, Tokyo 113-0033, Japan.

[7]Laboratory of Immunology, Institute for Frontier Life and Medical Sciences, Kyoto University, Kyoto 606-8507, Japan

[8]Graduate School of Frontier Science, The University of Tokyo, Chiba 277-8562, Japan

[9]Department of Integrative Bioanalytics, Institute of Development, Aging and Cancer (IDAC), Tohoku University, Sendai 980-8575, Japan.

*Corresponding Author:
Shinpei Kawaoka, Ph.D.
Department of Integrative Bioanalytics
Institute of Development, Aging and Cancer (IDAC)
Tohoku University
4-1 Seiryo-machi, Aoba-ku, Sendai 980-8575, Japan
TEL: +81-22-717-8568
Email: kawaokashinpei@gmail.com

Inter-Organ Communication Research Team
Institute for Life and Medical Sciences
Kyoto University
53 Shogoin-kawahara-cho, Sakyo-ku, Kyoto 606-8507, Japan
TEL: +81-75-751-4804
FAX: +81-75-751-3839
Email: kawaokashinpei@gmail.com

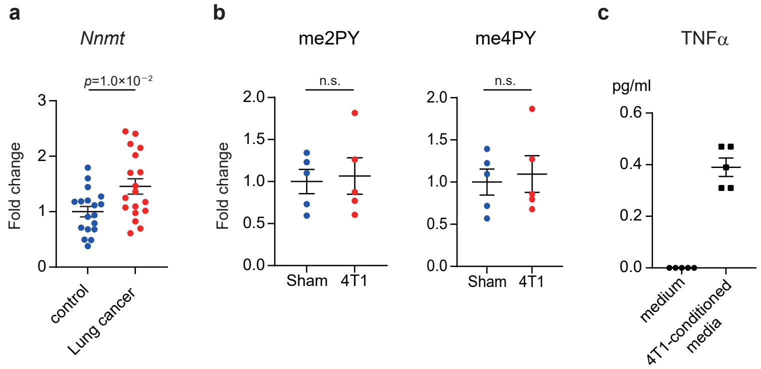

Fig. S1 Solid cancers up-regulate *Nnmt* expression in the liver

a. Expression of *Nnmt* in the livers of genetically induced lung cancer-bearing mice. Averaged fold change scores to the control group are shown presented as the mean ± SEM. The data are retrieved from the previous study[1]. n = 18. The exact p value is shown (unpaired two-tailed Student's t-test).

b. LC-MS/MS analysis for me2PY and me4PY in the livers of WT 4T1-bearing mice. n = 5. Averaged fold change scores to the sham-operated mice are presented as the mean ± SEM. n.s., not significant, unpaired two-tailed Student's t-test.

c. TNFα measurements (pg/ml) from the 4T1-conditioned media. n = 5.

Source data are provided as a source data file.

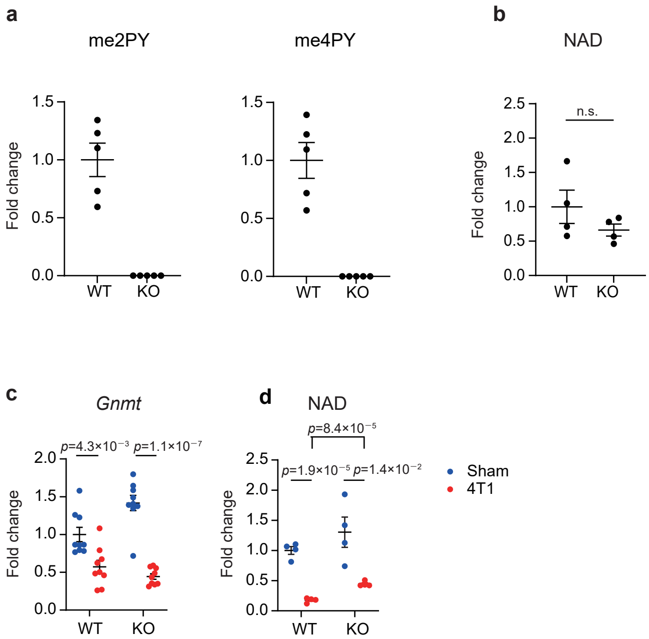

Fig. S2 Effects of 4T1 transplantation and *Nnmt* KO in liver metabolism

a. LC-MS/MS analysis for me2PY and me4PY in the livers of WT and *Nnmt* KO mice. n = 5.

b. LC-MS/MS analysis for NAD in the livers of WT and *Nnmt* KO mice. n = 4. n.s., not significant, unpaired two-tailed Student's t-test.

c. qPCR analysis for *Gnmt* in the livers of sham-operated mice and 4T1-bearing mice in WT and *Nnmt* KO. n = 9. The exact p values are shown (unpaired two-tailed Student's t-test).

d. LC-MS/MS analysis for NAD in the livers of sham-operated mice and 4T1-bearing mice in WT and *Nnmt* KO. n = 4. The exact p values are shown (unpaired two-tailed Student's t-test).

Averaged fold change data to the cancer-free WT mice are presented as the mean ± SEM.

Source data are provided as a source data file.

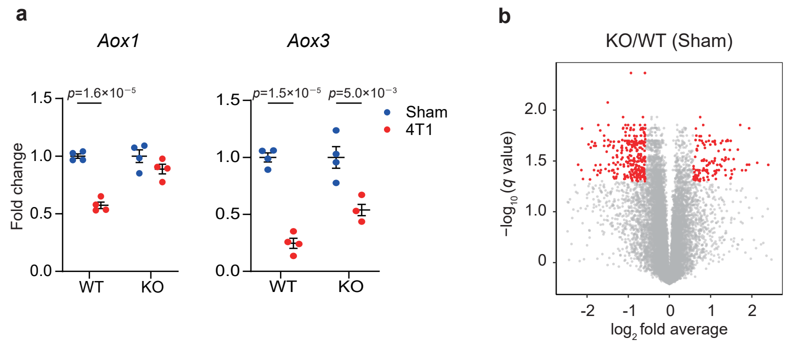

Fig. S3 Effects of *Nnmt* KO in hepatic gene expression

a. RNA-seq analysis for *Aox1* and *Aox3* in the livers of 4T1-bearing mice in WT and *Nnmt* KO (14 days after 4T1 transplantation). The exact *p* values are shown (unpaired two-tailed Student's *t*-test). $n = 4$. Averaged fold change data normalized to the sham group in each genotype are presented as the mean ± SEM.

b. RNA-seq experiments from the livers of sham-operated WT and *Nnmt* KO mice. A volcano plot ($\log_2$ fold average (KO/WT) versus $-\log_{10}$ (q value)) is shown. Genes showing more than 1.5-fold change with $q < 0.05$ are highlighted in red. $n = 4$.

Source data are provided as a source data file.

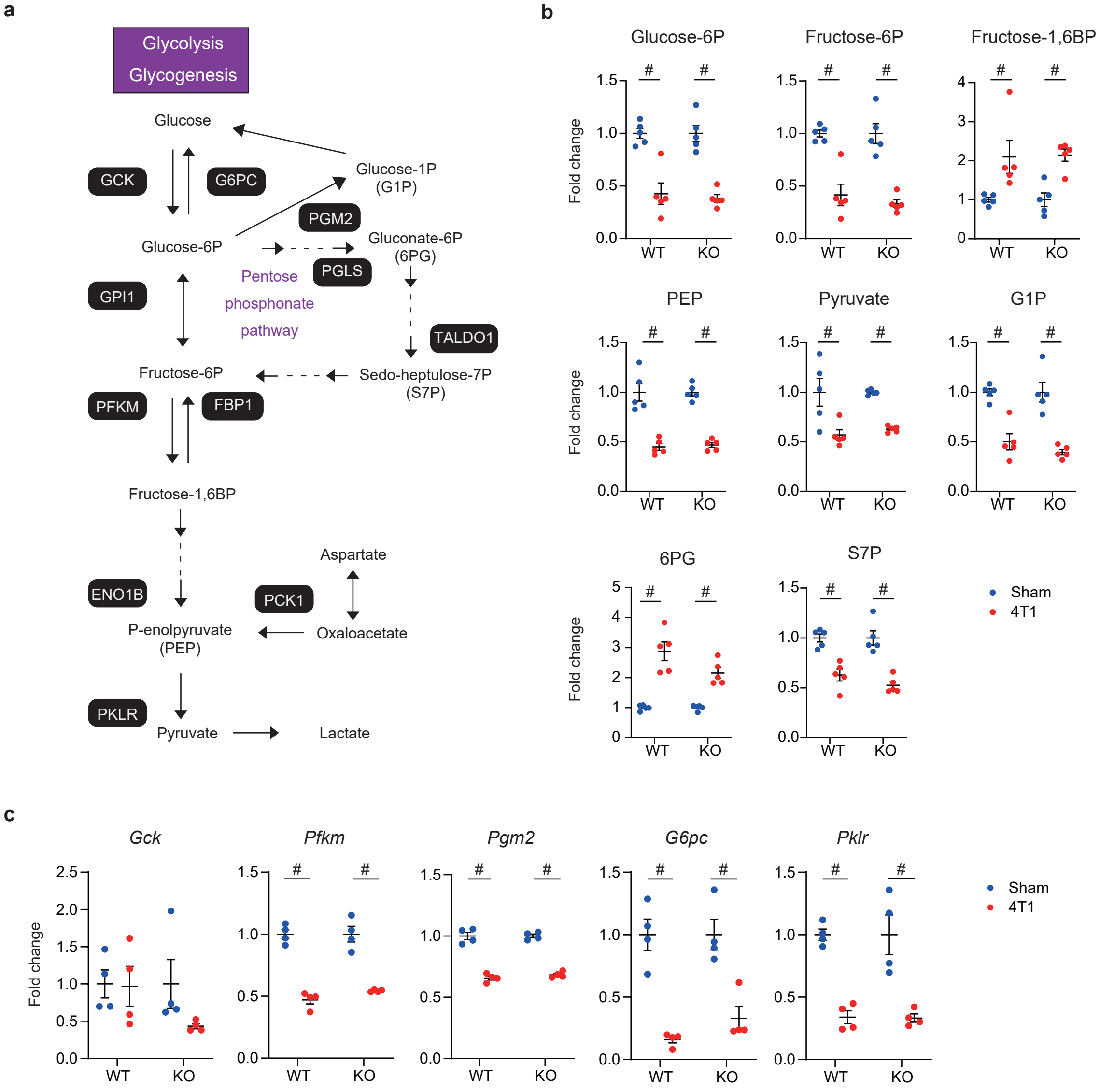

Fig. S4 Effects of *Nnmt* KO in 4T1-induced changes in glucose metabolism in the liver

a. Glucose metabolism pathways.

b. Expression of glucose pathway metabolites shown in **a**. $n = 5$.

c. RNA-seq results of genes encoding glucose metabolism enzymes shown in **a**. $n = 4$.

Averaged fold change data normalized to the sham group in each genotype are presented as the mean ± SEM.

#; more than 1.5-fold change with $q < 0.05$.

Source data are provided as a source data file.

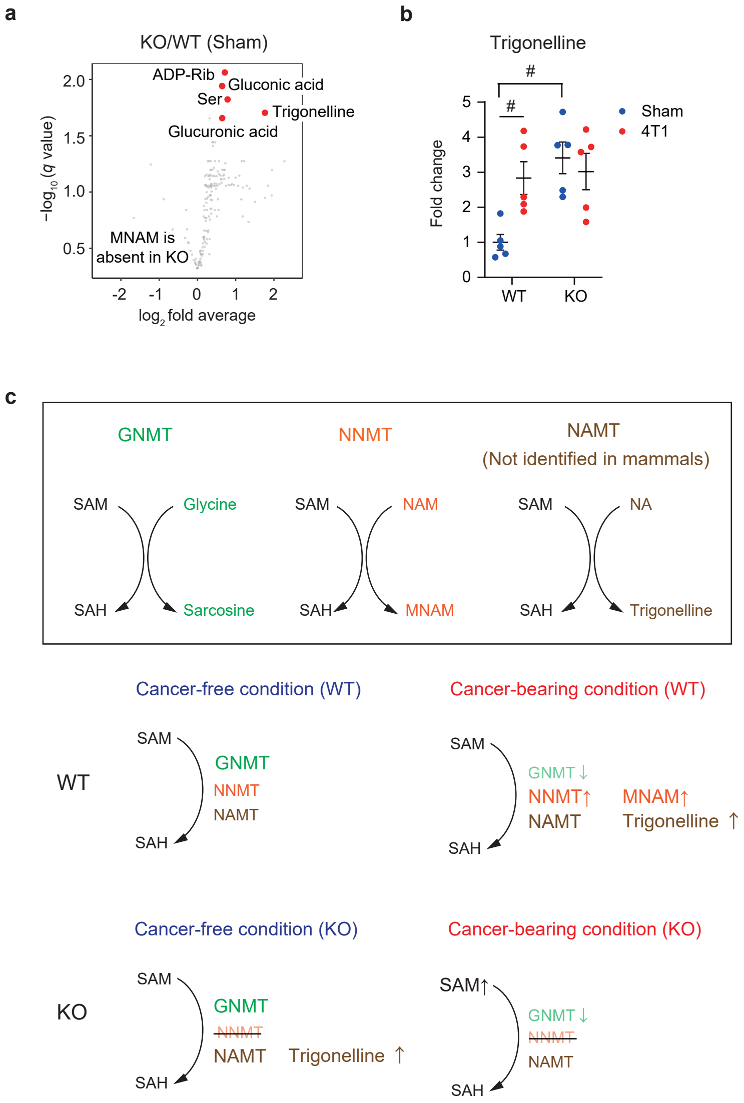

Fig. S5 A complexly rewired methyl-donor balance in the livers of 4T1-bearing mice

a. Metabolome experiments from the livers of sham-operated WT and *Nnmt* KO mice. A volcano plot ($\log_2$ fold average (KO/WT) versus $-\log_{10}$ (q value)) of WT (left) and *Nnmt* KO (right) are shown. Metabolites showing more than 1.5-fold change with $q < 0.05$ are highlighted in red. $n = 5$.

b. LC-MS/MS analysis for trigonelline. Averaged fold change data normalized to the sham group in WT are presented as the mean ± SEM. #; > 1.5-fold change with $q < 0.05$. $n = 5$.

c. A model that explains altered methyl-donor balance in the livers of 4T1-bearing mice. In the cancer-free WT livers, GNMT plays the major role in consuming SAM. In the 4T1-bearing condition, *Gnmt* is decreased while *Nnmt* and *Namt* are elevated. This possibly compensates the *Gnmt* down-regulation. Indeed, *Nnmt* KO in the presence of cancers increases SAM. In the cancer-free condition, it is likely that the enhanced trigonellin production consumes SAM that is leftover due to the loss of *Nnmt*. Such interaction among *Gnmt*, *Nnmt*, and *Namt* in the liver may play important roles in maintaining the methyl-donor balance.

Source data are provided as a source data file.

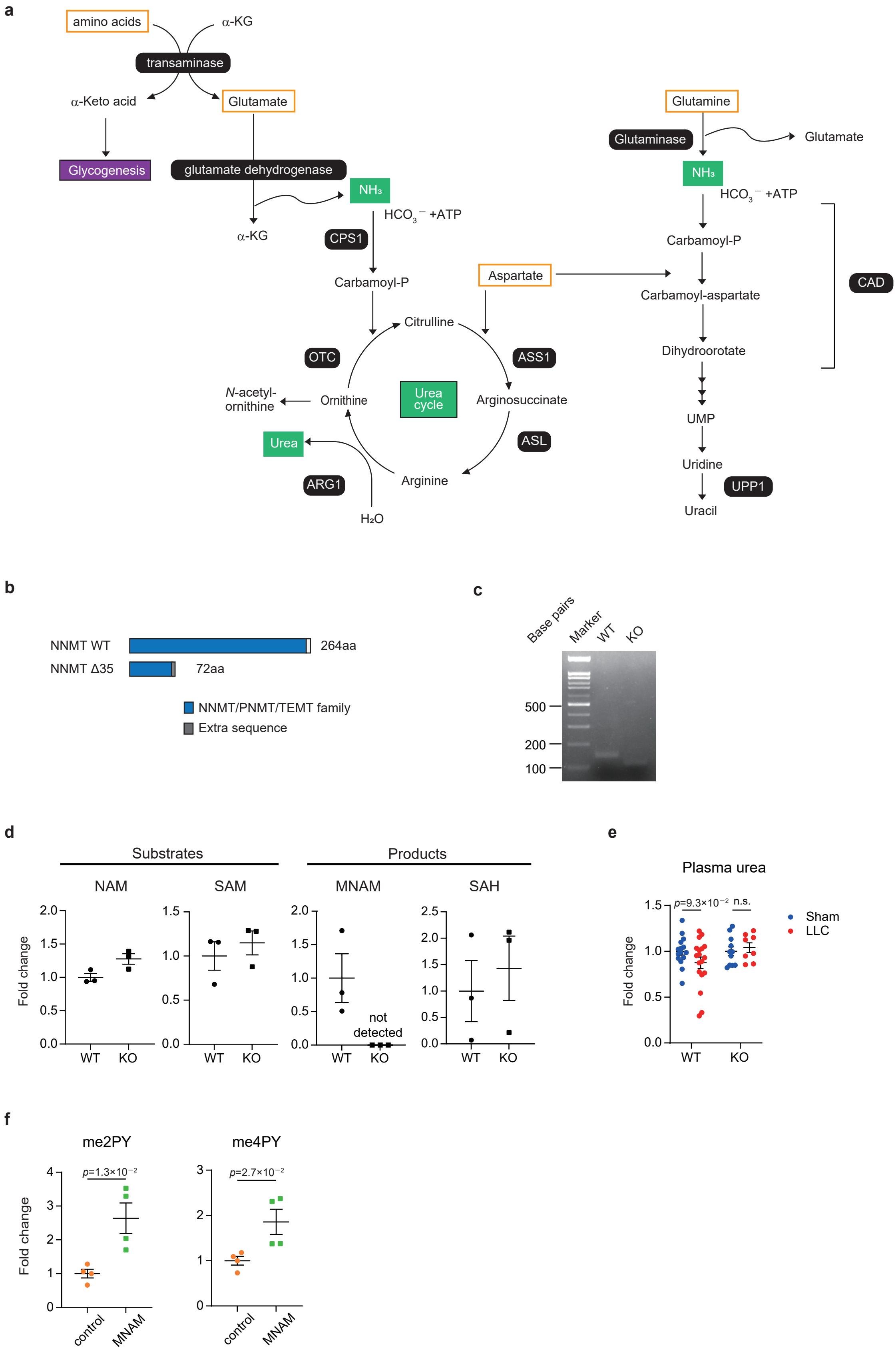
a
amino acids
α-KG
transaminase
α-Keto acid
Glutamate
Glycogenesis
glutamate dehydrogenase
NH₃
α-KG
HCO₃⁻ +ATP
CPS1
Carbamoyl-P
Aspartate
Citrulline
OTC
ASS1
N-acetyl-ornithine
Ornithine
Urea cycle
Arginosuccinate
Urea
ASL
ARG1
Arginine
H₂O
Glutamine
Glutaminase
Glutamate
NH₃
HCO₃⁻ +ATP
Carbamoyl-P
Carbamoyl-aspartate
CAD
Dihydroorotate
UMP
Uridine
UPP1
Uracil
b
NNMT WT
264aa
NNMT Δ35
72aa
NNMT/PNMT/TEMT family
Extra sequence
c
Base pairs
Marker
WT
KO
500
200
100
d
Substrates
Products
NAM
SAM
MNAM
SAH
Fold change
not detected
WT
KO
e
Plasma urea
p=9.3×10⁻²
n.s.
Sham
LLC
Fold change
WT
KO
f
me2PY
me4PY
p=1.3×10⁻²
p=2.7×10⁻²
Fold change
control
MNAM

Fig. S6 Analyses of C57BL/6N *Nnmt* KO mice

a. The urea cycle and uracil biogenesis. Nitrogen sources (aspartate and glutamine) are highlighted in orange.

b. The *Nnmt* KO (Δ35 allele) generated by the CRISPR-Cas9 technique in this study.

c. The representative picture for genotyping C57BL/6N *Nnmt* KO mice.

d. LC-MS/MS analysis for the NNMT-related metabolites in the livers of WT and *Nnmt* KO mice. n = 3.

e. The plasma urea level measured on 14 days after LLC transplantation. n.s., not significant, unpaired two-tailed Student's t-test. The exact p value is also shown. n = 14 for sham-operated WT mice, n = 18 for LLC-bearing WT mice, n = 9 for sham-operated *Nnmt* KO mice, n = 8 for LLC-bearing *Nnmt* KO mice.

f. LC-MS/MS analysis for me2PY and me4PY in the livers. Mice are injected with MNAM (250 mg/kg MNAM) in a daily manner for 12 days. n = 4. The exact p values are shown (unpaired two-tailed Student's t-test).

Averaged data normalized to the control group are presented as the mean ± SEM.

Source data are provided as a source data file.

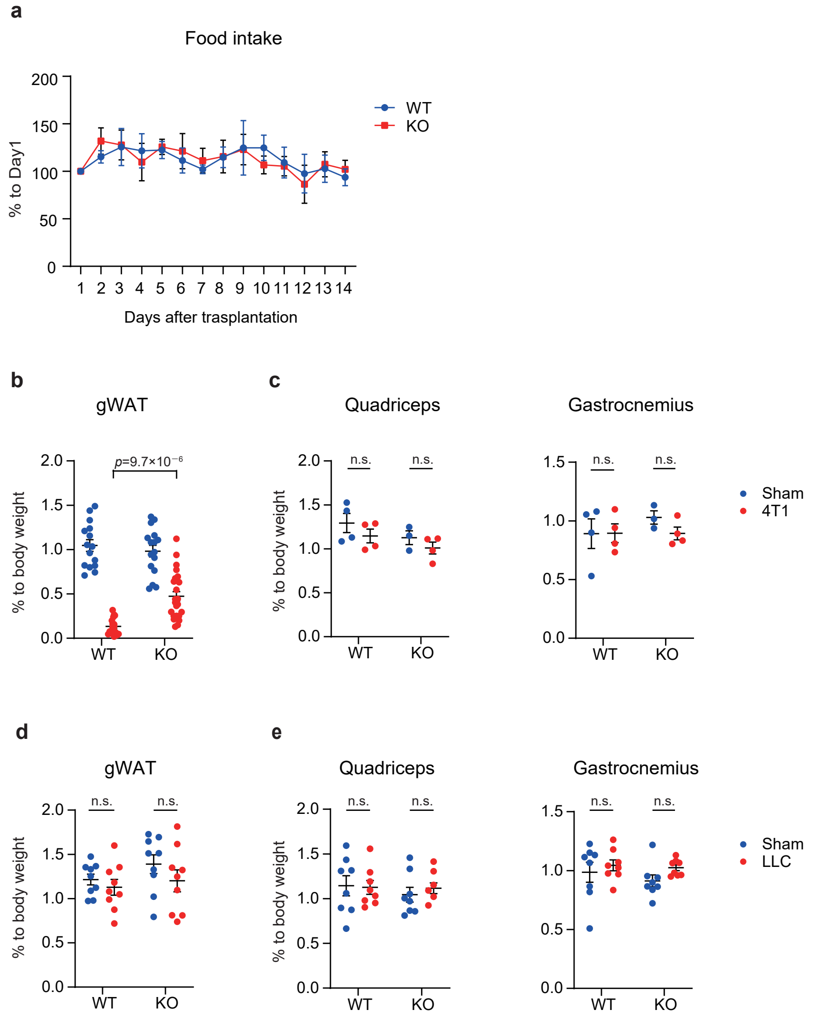

Fig. S7 Food intake, adipose tissues, and muscle tissues in 4T1-bearing mice

a. Food intake measured on 1-14 days after 4T1 transplantation in WT and *Nnmt* KO. Relative food intake is shown as % to the food intake on Day1. n = 3.

b. Changes in gonadal white adipose tissue (gWAT) weight measured on D14 after 4T1-transplantation in WT and *Nnmt* KO. n = 15 for sham-operated WT mice, n = 17 for 4T1-bearing WT mice, n = 16 for sham-operated *Nnmt* KO mice, n = 25 for 4T1-bearing *Nnmt* KO mice.

c. Quadriceps and gastrocnemius muscle weight measured on D14 after 4T1-transplantation in WT and *Nnmt* KO. n = 3 for sham-operated *Nnmt* KO mice. n = 4 for the other three experimental groups.

d. Changes in gWAT weight measured on D14 after LLC-transplantation in WT and *Nnmt* KO. n = 9.

e. Quadriceps and gastrocnemius muscle weight measured on D14 after LLC-transplantation in WT and *Nnmt* KO. n = 8.

b-e. Data are normalized to body weight (%). n.s., not significant, the exact p values are shown (unpaired two-tailed Student's t-test). Data are presented as the mean ± SEM.

Source data are provided as a source data file.

Supplementary References

1. Masri, S. et al. Lung Adenocarcinoma Distally Rewires Hepatic Circadian Homeostasis. Cell 165, 896-909, doi:10.1016/j.cell.2016.04.039 (2016).
