# Supplementary material for: Remote solid cancers rewire hepatic nitrogen metabolism via host nicotinamide-N-methyltransferase
Source: Nat Commun. 2022 Jun 15;13:3346. doi: 10.1038/s41467-022-30926-z (PMC9200709; doi:10.1038/s41467-022-30926-z)
Supplement: Supplementary file 3 — Description of Additional Supplementary Files [file 41467_2022_30926_MOESM3_ESM.pdf]

Description of Additional Supplementary Files

Supplementary Data 1: Primer and guide RNA oligo sequences used in this study.

Supplementary Data 2: RPKM scores from the livers of sham-operated mice and 4T1-bearing mice in WT and *Nnmt* KO. $n = 4$.

Supplementary Data 3: The quantitative content of metabolites from the livers of sham-operated mice and 4T1-bearing mice in WT and *Nnmt* KO. $n = 5$.
