# Supplementary material for: Remote solid cancers rewire hepatic nitrogen metabolism via host nicotinamide-N-methyltransferase
Source: Nat Commun. 2022 Jun 15;13:3346. doi: 10.1038/s41467-022-30926-z (PMC9200709; doi:10.1038/s41467-022-30926-z)
Supplement: Supplementary file 7 — Reporting Summary [file 41467_2022_30926_MOESM7_ESM.pdf]

nature portfolio

Corresponding author(s): Shinpei Kawaoka

Last updated by author(s): May 17, 2022

Reporting Summary

Nature Portfolio wishes to improve the reproducibility of the work that we publish. This form provides structure for consistency and transparency in reporting. For further information on Nature Portfolio policies, see our Editorial Policies and the Editorial Policy Checklist.

Statistics

For all statistical analyses, confirm that the following items are present in the figure legend, table legend, main text, or Methods section.

n/a	Confirmed	
☐	☒	The exact sample size (n) for each experimental group/condition, given as a discrete number and unit of measurement
☐	☒	A statement on whether measurements were taken from distinct samples or whether the same sample was measured repeatedly
☐	☒	The statistical test(s) used AND whether they are one- or two-sided<br>*Only common tests should be described solely by name; describe more complex techniques in the Methods section.*
☒	☐	A description of all covariates tested
☐	☒	A description of any assumptions or corrections, such as tests of normality and adjustment for multiple comparisons
☐	☒	A full description of the statistical parameters including central tendency (e.g. means) or other basic estimates (e.g. regression coefficient) AND variation (e.g. standard deviation) or associated estimates of uncertainty (e.g. confidence intervals)
☐	☒	For null hypothesis testing, the test statistic (e.g. F, t, r) with confidence intervals, effect sizes, degrees of freedom and P value noted<br>*Give P values as exact values whenever suitable.*
☒	☐	For Bayesian analysis, information on the choice of priors and Markov chain Monte Carlo settings
☒	☐	For hierarchical and complex designs, identification of the appropriate level for tests and full reporting of outcomes
☒	☐	Estimates of effect sizes (e.g. Cohen's d, Pearson's r), indicating how they were calculated

Our web collection on statistics for biologists contains articles on many of the points above.

Software and code

Policy information about availability of computer code

Data collection	LabSolutions
Data analysis	Feedam-BMPC software, Excel, GraphPad Prism 9, LabSolutions, and R were used for data analysis

For manuscripts utilizing custom algorithms or software that are central to the research but not yet described in published literature, software must be made available to editors and reviewers. We strongly encourage code deposition in a community repository (e.g. GitHub). See the Nature Portfolio guidelines for submitting code & software for further information.

Data

Policy information about availability of data

All manuscripts must include a data availability statement. This statement should provide the following information, where applicable:
- Accession codes, unique identifiers, or web links for publicly available datasets
- A description of any restrictions on data availability
- For clinical datasets or third party data, please ensure that the statement adheres to our policy

The RNA-seq data have been deposited in DNA Data Bank of Japan (DDBJ) under the accession code DRA011922 (https://ddbj.nig.ac.jp/resource/sra-submission/DRA011922). The metabolomics data have been deposited in Metabolomics Workbench (https://www.metabolomicsworkbench.org/) under the study IDs of ST002163 and ST002167). All other data are included as the supplementary information.

Field-specific reporting

Please select the one below that is the best fit for your research. If you are not sure, read the appropriate sections before making your selection.

☒ Life sciences ☐ Behavioural & social sciences ☐ Ecological, evolutionary & environmental sciences

For a reference copy of the document with all sections, see nature.com/documents/nr-reporting-summary-flat.pdf

Life sciences study design

All studies must disclose on these points even when the disclosure is negative.

Sample size	The sample size was empirically determined depending on the size effects. The number of animals was minimized as much as possible in light of animal ethics. In most cases, n = 4-5 was set as a threshold(Krzywinski, M. & Altman, N. Points of significance: Comparing samples-part I. Nat Methods 11, 215-216 (2014)). Dot-plot representation of data was exploited to obtain insights into how the samples were distributed and thus into the extent of difference of two groups.
Data exclusions	No data exclusion was performed.
Replication	Most experiments except omics measurements (RNA-seq and metabolome) were performed at least twice with multiple biological replicates, resulting in successful confirmation. Omics experiments were performed once with multiple biological replicates, and the results were validated using different experimental measures.
Randomization	Mice were randomly assigned to different experimental groups (e. g., sham and cancer transplantation). We did not have any specific criteria to allocate mice into experimental groups.
Blinding	No blinding was done. In the cancer transplantation experiments, the existence of cancers is obvious by the eye. Blinding mouse genotypes were not possible because the same researchers worked on genotyping and cancer transplantation experiments.

Reporting for specific materials, systems and methods

We require information from authors about some types of materials, experimental systems and methods used in many studies. Here, indicate whether each material, system or method listed is relevant to your study. If you are not sure if a list item applies to your research, read the appropriate section before selecting a response.

Materials & experimental systems

n/a	Involved in the study	
☒	☐	Antibodies
☐	☒	Eukaryotic cell lines
☒	☐	Palaeontology and archaeology
☐	☒	Animals and other organisms
☒	☐	Human research participants
☒	☐	Clinical data
☒	☐	Dual use research of concern

Methods

n/a	Involved in the study	
☒	☐	ChIP-seq
☒	☐	Flow cytometry
☒	☐	MRI-based neuroimaging

Eukaryotic cell lines

Policy information about cell lines

Cell line source(s)	4T1, Colon26, ID8-F3, LLC, and AML12.
Authentication	4T1 and Colon26 were provided by Dr. T. Nojiri. Expression markers were confirmed for 4T1 using RNA-seq. IF8-F3 was provided by Dr. McNeish. LLC was from the RIKEN BioResourse Center, Japan. AML12 was from American Type Culture Collection (ATCC, VA, USA).AML12 cells were authenticated analyzing Albumin gene expression. Colon26, IF8-F3, and LLC were not molecularly authenticated in our hands.
Mycoplasma contamination	The above-described cell lines except for AML12 before the experiments were proven free from mycoplasma contamination. AML12 cells were not tested for mycoplasma contamination.
Commonly misidentified lines (See ICLAC register)	The cell lines used are not listed as commonly misidentified cell lines.

Animals and other organisms

Policy information about studies involving animals; ARRIVE guidelines recommended for reporting animal research

Laboratory animals	8-10-week-old BALB/c females, BALB/c males, and C57BL/6N males. We have provided the other required information in our manuscript.
Wild animals	No wild animals were used in this study.

Field-collected samples	No field collected samples were used in this study.
Ethics oversight	All animal experiment protocols were approved by the Animal Care and Use committee of Advanced Telecommunications Research Institute International (April 2014 to October 2018) and that of Kyoto University (October 2018 to current).

Note that full information on the approval of the study protocol must also be provided in the manuscript.
